# Physiological Oxygen Levels in the Microenvironment Program Ex Vivo-Generated Conventional Dendritic Cells Toward a Tolerogenic Phenotype

**DOI:** 10.3390/cells14100736

**Published:** 2025-05-18

**Authors:** Antonia Peter, Morgane Vermeulen, Mats Van Delen, Amber Dams, Stefanie Peeters, Hans De Reu, Waleed F. A. Marei, Zwi N. Berneman, Nathalie Cools

**Affiliations:** 1 Laboratory of Experimental Hematology, Vaccine & Infectious Disease Institute (VAXINFECTIO), Faculty of Medicine and Health Sciences, University of Antwerp, 2610 Antwerp, Belgium; 2 Health Department, Flemish Institute for Technological Research (VITO), 2400 Mol, Belgium; 3 Center for Cell Therapy and Regenerative Medicine, Antwerp University Hospital, 2650 Edegem, Belgium; 4 Flow Cytometry and Sorting Core Facility (FACSUA), University of Antwerp, 2610 Antwerp, Belgium; 5 Gamete Research Centre, Laboratory of Veterinary Physiology and Biochemistry, Department of Veterinary Sciences, University of Antwerp, 2610 Antwerp, Belgium; 6 Department of Theriogenology, Faculty of Veterinary Medicine, Cairo University, Giza 3725005, Egypt

**Keywords:** dendritic cells, microenvironment, oxygen, immunometabolism, dendritic-cell based immunotherapy

## Abstract

Dendritic cells (DCs) are critical regulators of immune homeostasis, balancing tolerance and immunity through antigen presentation and T cell modulation. While the influence of hypoxia (<2% O_2_) on DC function in pathological settings is well-documented, the impact of physiological O_2_ levels remains underexplored. This study investigates the role of physioxia (4% O_2_) in programming mature DCs toward a tolerogenic phenotype compared to atmospheric conditions (21% O_2_) typically present in in vitro assays. DC cultures generated under 4% O_2_ exhibited a reduced monocyte-to-DC transformation rate, increased lactate production, a semi-mature surface marker profile, and increased surface expression of the tolerance-associated marker ILT4. T cell priming was altered only when atmospheric DCs were co-cultured under physioxia, suggesting an O_2_-dependent threshold for immunostimulatory capacity. These findings highlight the complexity of O_2_-dependent mechanisms in DC-T cell interactions, revealing a delicate balance between tolerance and immunogenicity. Our results underscore the need for physiologically relevant O_2_ conditions in DC research to better reflect in vivo behavior and inform immunotherapy design. Overall, this study advances understanding of how microenvironmental cues shape DC biology, with implications for immune tolerance, autoimmunity, and cancer immunotherapy.

## 1. Introduction

Dendritic cells (DCs) are professional antigen-presenting cells that serve as a critical link between the innate and adaptive immune systems. Derived from CD34^+^ bone marrow-resident hematopoietic stem cells, they are strategically distributed throughout the body to perform immune surveillance and modulate immune responses [1,2,3]. Under homeostatic conditions, DCs capture self-antigens and harmless environmental antigens (e.g., commensal microbes, apoptotic cells) in peripheral tissues such as the skin, lungs, and gut [4]. Immature (i) DCs, characterized by high endocytic capacity, low antigen presentation capacity, low expression of co-stimulatory molecules, and minimal cytokine secretion, present these antigens in a non-inflammatory context to T cells in secondary lymphoid organs [5,6,7,8]. This process promotes peripheral tolerance by inducing regulatory T cells (Tregs) or silencing self-reactive T cells, thereby preventing autoimmune responses. During inflammation, danger signals such as damage-associated molecular patterns (DAMPs) or pathogen-associated molecular patterns (PAMPs) trigger DCs into maturation [9,10,11]. This involves upregulation of antigen presentation via the major histocompatibility complex, co-stimulatory molecules (e.g., CD80, CD86), and chemokine receptors, enabling their efficient migration to secondary lymphoid organs [12,13,14]. Mature (m)DCs subsequently present processed antigens to T cells, initiating adaptive immune responses tailored to the detected pathogen [15,16]. Depending on the specific microenvironmental signals they receive, mDCs can promote tolerance or immunogenicity [17,18,19,20]. For example, in a tolerogenic context, inhibitory signals such as the programmed death-ligand (PD-L)1/2—programmed death-1 (PD-1), CD155—T cell immunoreceptor with immunoglobulin and ITIM domains (TIGIT), and CD80/CD86—cytotoxic T lymphocyte-associated protein-4 (CTLA-4) interactions, combined with secretion of anti-inflammatory cytokines (e.g., IL-10), suppress effector T cell activation and support Treg induction [21,22,23,24]. In an immunogenic context, CD80 and CD86 interact with CD28 on T cells and, in combination with pro-inflammatory cytokines like IL-12, drive effector T cell activation and differentiation, promoting robust immune responses to pathogens [25,26]. Through the integration of cytokine secretion profiles and surface marker expression, mDCs can prime Tregs for immune tolerance or activate T helper cell subsets such as Th1, Th2, and Th17 [27,28]. These subsets coordinate responses against intracellular pathogens, extracellular parasites, or mucosal infections, respectively. The ability of DCs to balance tolerance and immunogenicity is crucial for maintaining immune homeostasis, as an overactive response may lead to allergies or autoimmunity, whereas insufficient activation could result in uncontrolled infections or tumor progression. Homeostatic immune responses aim to prevent unwanted immune activation while maintaining vigilance for potential threats.

These diverse functional outcomes are mediated by different DC subsets, each of which plays a specialized role in shaping immune responses. DC subsets include conventional (c)DCs, plasmacytoid DCs, and monocyte-derived (mo)DCs [29]. cDCs are further classified into cDC1 and cDC2 based on their lineage and function, with cDC1 excelling in cross-presentation and cytotoxic T cell activation, while cDC2 specializes in activating T helper cells. pDCs play a critical role in antiviral immunity through the production of type I interferons, enhancing the immune response to viral infections. moDCs, often generated ex vivo for autologous immunotherapies, can also differentiate from monocytes in vivo under inflammatory conditions, highlighting the plasticity of the immune system and its ability to adapt to pathological states. Together, these subsets provide the functional versatility required for DCs to balance immune responses across diverse physiological and pathological scenarios.

In both health and disease, the tissue microenvironment plays a crucial role in guiding immune functions. The microenvironment is composed of various factors, including cell interactions, cytokines, nutrients, metabolites, pH, and oxygen (O_2_) levels, all of which collectively influence cellular behavior and function [30,31,32]. In a homeostatic state, these components are tightly regulated to maintain normal tissue function and immune balance. However, in disease states such as cancer, chronic inflammation, and ischemic conditions, homeostatic mechanisms become dysregulated. This dysregulation imposes significant metabolic and physiological challenges, leading to altered immune responses and pathological outcomes [33,34,35,36]. Among the various factors in the tissue microenvironment, O_2_ availability plays a pivotal role in shaping the metabolic and functional states of immune cells, including DCs. Physiological O_2_ levels range from 4–14% in the blood, 3–9% in well-vascularized tissues, and as low as 0.5% in lymphoid organs [37,38,39,40,41]. In contrast, atmospheric O_2_ levels (21%) typically used in cell culture represent a hyperoxic state and do not reflect in vivo environments [41,42,43,44,45,46,47,48]. While the role of O_2_ in the regulation of DC function has been extensively studied in pathological states such as tumors, wounds, and inflamed tissues, characterized by hypoxia (<2%) [49], its specific role in immune homeostasis remains underexplored.

This study investigates whether physiological O_2_ levels (4%) program DCs toward a tolerogenic phenotype, thereby contributing to immune homeostasis. To explore the role of O_2_ in DC biology, we applied 4% O_2_ during the DC generation period to mimic the O_2_ conditions typically encountered in peripheral tissues. We assessed the resulting cells in terms of their phenotype, mitochondrial function, and capacity to activate allogeneic T cells. Recognizing and incorporating physiologically relevant O_2_ conditions into experimental designs may enhance our understanding of how DCs operate in their natural microenvironment and support the clinical translation of DC-based therapies. Ultimately, gaining deeper insights into how O_2_ shapes DC biology will advance our understanding of immune responses in both health and disease.

## 2. Materials and Methods

### 2.1. Cells and Culture Conditions

**Figure 1:**
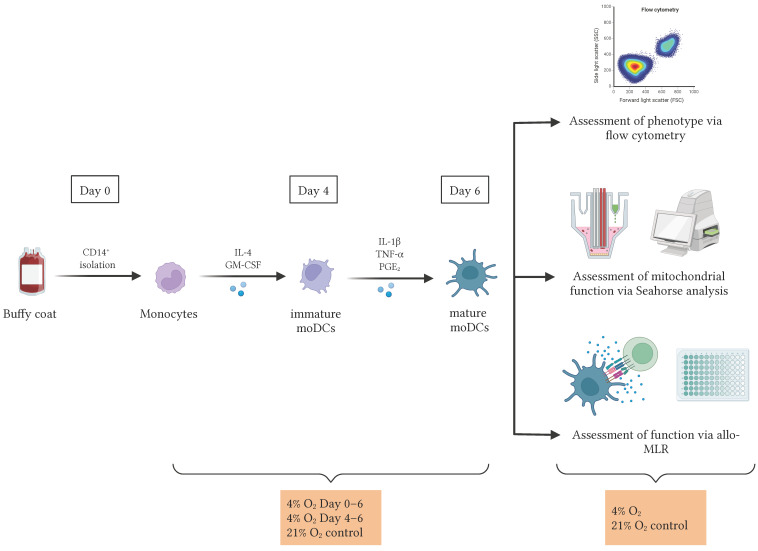
Workflow for the assessment of physioxic culture conditions in moDCs. DCs were differentiated from monocytes in the presence of IL-4 and GM-CSF and matured with the pro-inflammatory cytokines IL-1β, TNF-α, and PGE_2_. Cultures were divided into three experimental groups: (1) continuous culture under 21% O_2_ (atmospheric O_2_ levels), (2) continuous culture under 4% O_2_ (days 0–6), and (3) 4% O_2_ during the maturation phase only (days 4–6). The latter was included to provide a reference for late-stage low-O_2_ exposure and to support interpretation of the sustained low-O_2_ exposure group. On day 6, cell phenotype was analyzed by flow cytometry, mitochondrial function was assessed using a Seahorse assay, and immunostimulatory capacity was evaluated in an allo-MLR performed at either 4% or 21% O_2_. This setup was designed to better mimic the physiological O_2_ levels found in human tissues. Abbreviations used: moDCs, monocyte-derived dendritic cells; IL, interleukin; GM-CSF, granulocyte-macrophage colony-stimulating factor; TNF-α, tumor necrosis factor-alpha; PGE_2_, prostaglandin E_2_; O_2_, oxygen; allo-MLR, allogeneic mixed lymphocyte reaction.

Buffy coats derived from healthy donor whole blood collections were purchased from the Red Cross Flanders (Mechelen, Belgium) and processed as described previously [50], with each biological replicate, denoted as n, representing an independent donor. Peripheral blood mononuclear cells were isolated using Ficoll-Paque density gradient centrifugation (GE Healthcare, Diegem, Belgium) followed by isolation of CD14^+^ monocytes using CD14-microbead-based immunomagnetic separation (CD14 reagent; Miltenyi Biotec, Leiden, The Netherlands). To generate DCs, purified CD14^+^ cells were resuspended in Iscove’s Modified Dulbecco’s Medium (IMDM; Thermo Fisher Scientific, Merelbeke, Belgium), supplemented with 2% human AB serum (hAB; Thermo Fisher Scientific), 250 IU/mL interleukin (IL)-4 (Miltenyi Biotec), and 200 IU/mL granulocyte-macrophage colony-stimulating factor (GM-CSF; Gentaur, Kampenhout, Belgium) at day 0 at a density of 1 × 10^6^ cells/mL and cultured for a period of six days in culture flasks (Greiner Bio-One, Vilvoorde, Belgium). On day 4, a combination of pro-inflammatory cytokines, consisting of 1000 IU/mL IL-1β (Miltenyi Biotec), 1000 IU/mL tumor necrosis factor (TNF)-α (Miltenyi Biotec), and 2.5 µg/mL prostaglandin (PG)E_2_ (Pfizer, Puurs, Belgium) was added to the culture for a 48-h maturation stimulus. Cells were incubated at 37 °C in a humidified atmosphere with 5% CO_2_ under either 21% or 4% O_2_, using a Whitley H45 HEPA Hypoxystation (Don Whitley Scientific, Bingley, UK) for the latter (Figure 1). On day 6, mature moDCs were harvested for additional experiments.

The fraction of CD14^−^ cells, comprising peripheral blood lymphocytes (PBLs), was cryopreserved for further use at a concentration of 50 × 10^6^ cells/mL in fetal bovine serum (FBS; Thermo Fisher Scientific) supplemented with 10% dimethyl sulfoxide (DMSO; Merck Life Science, Hoeilaart, Belgium) and 4% glucose (Laboratoires Sterop, Brussel, Belgium). The aliquots were frozen in Corning CoolCell LX cell-freezing containers (Corning, Lasne, Belgium) at −80 °C.

### 2.2. Immunophenotyping of DCs

The quality of the cells was evaluated by assessing viability using flow cytometry with propidium iodide staining (Thermo Fisher Scientific) after the harvest on day 6. Additionally, DC surface marker expression was assessed through phenotypical analysis. Following a 5-min blocking step (Human TruStain FcX; BioLegend, Amsterdam, The Netherlands), cells were stained with two distinct antibody panels, both of which included a viability dye (LIVE/DEAD fixable Near-Infrared Dead Cell Stain; Thermo Fisher Scientific) and a TruStain monocyte blocker (BioLegend) to avoid unspecific binding of DCs to tandem fluorophores. The first panel was designed to assess DC identity and maturation, and contained the following fluorochrome-conjugated monoclonal antibodies: anti-CD209-phycoerythrin-cyanine7 (PE-Cy7; BioLegend), anti-human leukocyte antigen (HLA)-DR-Kiravia-Blue-520 (BioLegend), anti-CD14-Brilliant-Violet-711 (BV711; BioLegend), anti-CD83-BV785 (BioLegend), anti-CD80-allophycocyanin (APC, BioLegend), and anti-CD86-BV605 (BioLegend). The second panel focused on the analysis of tolerance-associated surface markers and included: anti-CD209-PE (BioLegend), anti-CD11c-PerCP/Cyanine5.5 (BioLegend), anti-B7 homolog 3 (B7-H3)-PE/Dazzle 594 (BioLegend), anti-programmed death-ligand 1 (PD-L1)-BV711 (BioLegend), anti-CD40-BV650 (BioLegend), and anti-immunoglobulin-like transcript 4 (ILT4)-APC (BioLegend). In addition, staining for ILT3 (anti-ILT3-PE; BioLegend) was performed separately in combination with the same viability dye. The concentrations used for each antibody are listed in Supplementary Table S1. Non-specific background staining was assessed using isotype-matched control antibodies or fluorescence-minus-one (FMO) controls, as appropriate. Compensations were set up with compensation beads (UltraComp eBeads Plus Compensation Beads, Thermo Fisher Scientific) according to the manufacturer’s instructions. Per condition, 1 × 10^5^ cells were washed in sheath buffer (BD FACSFlow Sheath Fluid, BD Biosciences, Erembodegem, Belgium) supplemented with 0.1% bovine serum albumin (BSA; Merck Life Science) and 0.05% sodium azide (Merck Life Science), stained in a volume of 100 µL for 15 min at 4 °C, and washed once more before data acquisition on a NovoCyte Quanteon flow cytometer (Agilent Technologies, Diegem, Belgium), collecting 10^4^ events per sample based on forward scatter (FSC) and side scatter (SSC) properties. All median fluorescence intensity (MFI) and percentage expression values were calculated from viable cells, as determined by exclusion of the L/D marker. Control populations consistently exhibited >99% CD209 and HLA-DR double-positivity, confirming their DC identity. Gating strategy and representative plots for all markers are provided in Supplementary Figure S1.

In addition, Mitotracker dyes were used for mitochondrial labeling of DCs: Mitotracker Green FM fluorescein isothiocyanate (FITC), Mitotracker Red FM CMXRos PE, and MitoSOX Red PE (Table S1). Mitochondrial labeling was performed in single stains in combination with a LIVE/DEAD fixable Near-Infrared Dead Cell Stain. All Mitotracker dyes are products of Thermo Fisher Scientific. Per condition, 1 × 10^5^ cells were washed in serum-free phosphate-buffered saline (PBS; Thermo Fisher Scientific), stained in a volume of 100 µL for 30 min at 37 °C, and washed once more before data acquisition as described above. MFI values were directly recorded from viable cells, gated as described in Supplementary Figure S1.

### 2.3. Lactate Measurement

To assess lactate production, 10 µL of cell culture supernatant was collected on days 2, 4, and 6 of the DC culture and promptly frozen at −20 °C until further analysis. Lactate concentrations were measured using a StatStrip Express Lactate device in combination with StatStrip Lactate test strips, according to the manufacturer’s instructions (Nova Biomedical, Boxtel, The Netherlands).

### 2.4. Seahorse Assay for Mitochondrial Function

Mitochondrial function was assessed on day 6 of DC culture using the Agilent Seahorse XFp Cell Mito Stress Test. Sensor cartridges were hydrated overnight with sterile water, starting the day before the assay. On the day of measurement, DCs were harvested and assessed for viability as described in Section 2.2. Hydrated sensor cartridges were then loaded with Seahorse XF Calibrant and incubated for at least 45 min at 37 °C in a non-CO_2_ incubator.

Meanwhile, DCs were resuspended in Seahorse XF RPMI assay medium (pH 7.4) supplemented with 10 mM glucose, 1 mM pyruvate, and 2 mM glutamine, and seeded into poly-D-lysine (PDL)-coated Seahorse XFp miniplates that had been pre-equilibrated to 37 °C in a non-CO_2_ incubator. Depending on the condition-specific viability, a total of 5 × 10^4^–2 × 10^5^ cells were plated per well. Plates were centrifuged briefly (300× *g*, 1 min) to ensure cell adherence.

Following drug loading of the sensor cartridges with 1.5 µM oligomycin (adenosine triphosphate (ATP) synthase inhibitor), 1.5 µM carbonyl cyanide-p-trifluoromethoxyphenylhydrazone (FCCP; uncoupling agent), and 0.5 µM rotenone/antimycin A (complex I/III inhibitors), the cartridges were placed into the Seahorse XF Mini Bioanalyzer for calibration. During this time, the cell plates were kept at 37 °C in a non-CO_2_ incubator. Once calibration was complete, the cell plate was inserted into the analyzer, and measurements of oxygen consumption rate (OCR) were taken before and after each compound injection. These were used to calculate key mitochondrial parameters reflecting different aspects of mitochondrial respiration, including basal respiration, ATP-linked respiration, proton leak, maximal respiration, spare respiratory capacity, non-mitochondrial respiration, and coupling efficiency. Each condition was tested in technical triplicates. Data were analyzed using the Agilent Seahorse Analytics software (version 1.0.0-749) and normalized to the number of total living DCs per well. All Seahorse-specific equipment and reagents were obtained from Agilent Technologies.

### 2.5. Allogeneic Mixed Lymphocyte Reaction (Allo-MLR)

To determine the T cell stimulatory capacity of DCs, allogeneic PBLs were co-cultured with DCs in a 10:1 ratio in 24-well plates (Greiner Bio-One), with the number of cells seeded based on live cell counts. PBLs stimulated with 1 µg/mL phytohaemagglutinin (PHA; Merck Life Science) served as a positive control. Furthermore, unstimulated allogeneic PBLs without DCs served as a negative control. Additionally, PBLs from the same donor as the DCs were included to control for potential effects of O_2_ deprivation on T cells in the absence of allogeneic stimulation (referred to as the PBL-only control in the results section). The co-cultures were performed in IMDM supplemented with 5% hAB and maintained for five days at 37 °C in a humidified atmosphere with 5% CO_2_ under either 21% or 4% O_2_ (Figure 1). After this incubation period, the supernatants were collected, frozen, and stored at −20 °C for subsequent testing. As a measure of the T cell stimulatory capacity of DCs, the levels of interferon (IFN)-γ were quantified using a commercially available enzyme-linked immunosorbent assay (ELISA; PeproTech, London, UK) according to the manufacturer’s instructions. Each sample was tested in triplicate. Plates were read by measuring absorbance at 405 nm using a Victor^3^ multilabel plate reader (PerkinElmer, Mechelen, Belgium) and interpolated to the concentration (pg/mL) using MS Office Excel.

### 2.6. Immunophenotyping of PBLs

To compare the stimulatory capacities of atmospheric and physioxic DCs to elicit allogeneic T cell responses not only based on IFN-γ secretion but also on the surface expression of co-stimulatory ligands and other markers, PBLs were stained with two different antibody panels on the last day of the allo-MLR following a 5-min blocking step (Human TruStain FcX). The first panel was used to distinguish different T cell subsets and contained the following fluorochrome-conjugated monoclonal antibodies: anti-CD45RA-Pacific-Blue, anti-CD8-BV510, anti-CD27-BV605, anti-CD57-BV785, anti-CD4-FITC, anti-CCR7-PE, anti-PD-1-PE-Cy7, anti-CD28-APC, anti-CD3-Spark-Red-718, and LIVE/DEAD fixable Near-Infrared Dead Cell Stain (Table S1). The second panel for T cell exhaustion markers contained the following fluorochrome-conjugated monoclonal antibodies: anti-PD-L2-BV421, anti-CD8-BV510, anti-TIGIT-BV605, anti-TIM-3-BV711, anti-CTLA-4-BV785, anti-CD4-FITC, anti-CD96-PE, anti-LAG-3-PE-Cy7, anti-PD-L1-APC, anti-CD3-Spark-Red-718, and LIVE/DEAD fixable Near-Infrared Dead Cell Stain (Table S1). All antibodies are products of BioLegend, except for CCR7 (Bio-Techne, Abingdon, UK). Samples were prepared, stained, and acquired as described in Section 2.2, with 5 × 10^4^ live cells recorded per condition. Gating strategy and representative plots for all markers are provided in Supplementary Figure S2.

### 2.7. Statistical Analysis

Flow cytometric data were analyzed using the FlowJo 10.10.0 software (FlowJo, TreeStar Inc., Ashland, OR, USA). Values are presented as the median, unless otherwise indicated, with the interquartile range (IQR) expressed as median (Q25–Q75). Statistical analyses were performed using GraphPad Prism 10.10.2, with the respective tests outlined in the figure legends. Each dataset was initially tested for normality using the Shapiro-Wilk test. For comparisons between two groups, a two-tailed paired *t*-test was used for normally distributed data, and the Wilcoxon matched-pairs signed rank test for non-parametric analysis. When comparing three groups, data that did not pass the normality test were analyzed using the Kruskal-Wallis test with Dunn’s multiple comparisons due to the inability of the Friedman test for paired data to accommodate datasets with missing values. Normally distributed data were analyzed using a one-way ANOVA (non-paired) with Šídák’s multiple comparisons test. For analyses involving multiple levels of grouping, a mixed-effects model with the Geisser-Greenhouse correction and Šídák’s multiple comparisons test was employed. Volcano plots for the PBL phenotyping data were generated to visualize fold changes against the Benjamini-Hochberg-adjusted *p*-values derived from two-tailed paired *t*-tests. A fold change > 1.0 was classified as upregulation, while a fold change < 1.0 was classified as downregulation. A *p*-value < 0.05, corresponding to a −log (adjusted *p*-value) of 1.30, was considered statistically significant.

## 3. Results

### 3.1. Physiological O_2_ Levels During the Generation Period Resulted in a Decreased Transformation of Monocytes into DCs

**Figure 2:**
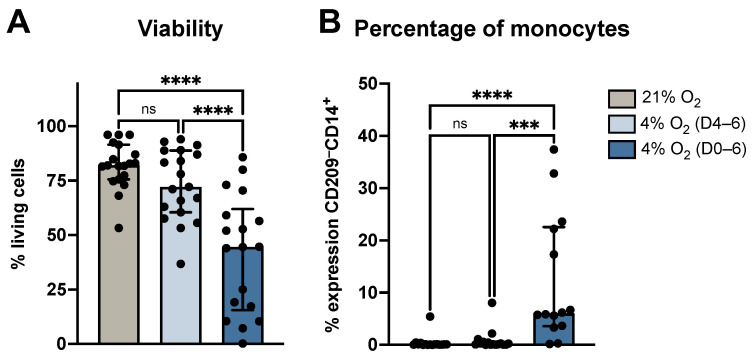
Generation of moDCs under 4% O_2_ resulted in decreased viability and increased percentage of monocytes. (**A**) Viability of the total cell population on day 6 determined via PI exclusion, prior to DC phenotyping (*n* = 20). (**B**) Monocyte population represented as the percentage of CD209^–^CD14^+^ cells (*n* = 16). All cells were cultured under atmospheric O_2_ levels, 4% O_2_ during the entire generation phase (D0–6), or 4% O_2_ only during the maturation phase (D4–6). Data are shown as median with interquartile range. Statistical analyses were performed using an ordinary one-way ANOVA (**A**) or the Kruskal-Wallis test with Dunn’s post-hoc analysis (**B**). Statistical significance is denoted by *, where *** represents *p* < 0.001 and **** represents *p* < 0.0001. Abbreviations used: moDCs, monocyte-derived dendritic cells; O_2_, oxygen; D0–6, days 0 to 6; D4–6, days 4 to 6; PI, propidium iodide; ANOVA, analysis of variance; ns, not significant.

To understand how O_2_ influences DC generation and phenotype under physiological conditions, we examined the impact of 4% O_2_, referred to hereafter as physioxia, on the generation of mature moDCs in comparison to 21% O_2_, which will be referred to as atmospheric O_2_ levels or control conditions. We employed two physioxic culture conditions: physioxia during the entire 6-day culture period (4% O_2_ days 0–6) and physioxia only during the maturation phase between days 4 and 6 (4% O_2_ D4–6), the latter serving as a maturation-phase-specific comparator to contextualize the effects of continuous low-O_2_ exposure. These were compared to continuous culture under 21% O_2_ (Figure 1). Our findings demonstrated a significant reduction in the monocyte-to-mDC transformation rate (control: 34.3%, IQR: 26.9–42.4%; 4% O_2_ D4–6: 33.4%, IQR: 31.4–40.4%; 4% O_2_ D0–6: 24.0%, IQR: 9.3–25.9%) under continuous physioxia compared to atmospheric O_2_ levels (*p* < 0.0001). This was calculated as the number of viable cells on day 6 excluding CD209^–^CD14^+^ monocytes, relative to the number of viable monocytes seeded on day 0. CD14 downregulation and CD209 upregulation was used to distinguish successfully transformed moDCs from undifferentiated monocytes, in line with established phenotypic criteria. The same effect was observed regarding cell viability (*p* < 0.0001) (Figure 2A). In contrast, no significant differences were observed when comparing control conditions to physioxic maturation conditions. Furthermore, the percentage of monocytes (CD209^–^CD14^+^) was significantly increased upon a full physioxic culture period compared to control conditions (*p* < 0.0001), whereas no such increase was observed when the cells were exposed to 4% O_2_ only during the maturation phase (Figure 2B). Taken together, these results suggest that physioxic culture conditions negatively affect DC generation from monocytes when applied throughout the entire culture period.

### 3.2. Physiological O_2_ Levels During the Generation Period Resulted in Decreased Surface Expression of Identity and Maturation Markers, and Increased Expression of Tolerance-Associated Marker ILT4

**Figure 3:**
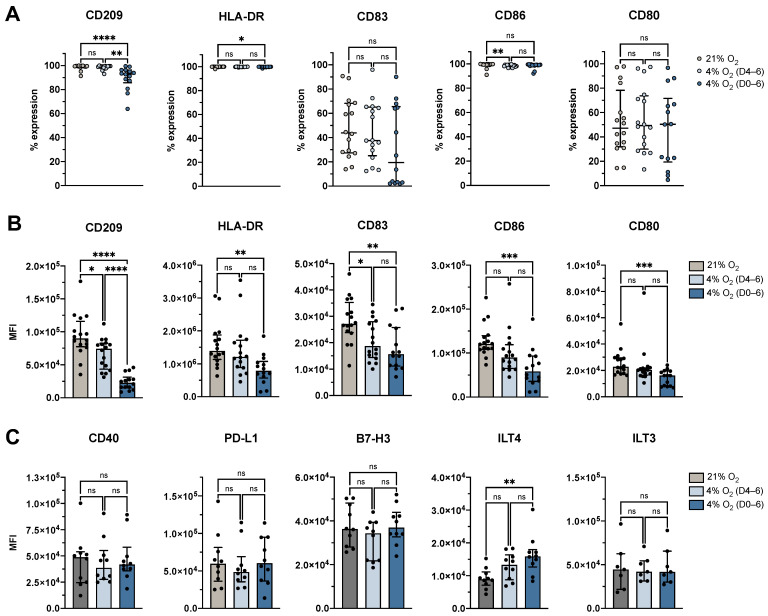
DC generation under 4% O_2_ resulted in a decreased surface expression of identity and maturation markers, and an increase of tolerance marker ILT4. (**A**) The percentage of expression for the markers CD209, HLA-DR, CD83, CD86, and CD80 (*n* = 16). (**B**) The expression levels of the markers CD209, HLA-DR, CD83, CD86, and CD80 presented as MFI of the positive population (*n* = 16). (**C**) The expression levels of the markers CD40, PD-L1, B7-H3, ILT4, and ILT3 presented as MFI of the positive population (*n* = 7 for ILT3, *n* = 10 for all other markers). Cells depicted in all graphs were cultured under atmospheric O_2_ levels, 4% O_2_ (D4–6), or 4% O_2_ (D0–6). Data are shown as median with interquartile range. Statistical analyses were performed using an ordinary one-way ANOVA ((**A**): CD83, CD80; (**B**): CD209, CD83; (**C**): CD40, B7-H3, ILT4, ILT3) or the Kruskal-Wallis test with Dunn’s post-hoc analysis ((**A**): CD209, HLA-DR, CD86; (**B**): HLA-DR, CD86, CD80; (**C**): PD-L1). Statistical significance is denoted by *, where * represents *p* < 0.05, ** represents *p* < 0.01, *** represents *p* < 0.001, and **** represents *p* < 0.0001. Abbreviations used: O_2_, oxygen; D0–6, days 0 to 6; D4–6, days 4 to 6; MFI, mean fluorescence intensity; ANOVA, analysis of variance; CD, cluster of differentiation; HLA-DR, human leukocyte antigen—DR isotype; PD-L1, programmed death-ligand 1; B7-H3, B7 homolog 3; ILT, immunoglobulin-like transcript; ns, not significant.

The generation of mDCs from monocytes is typically marked by downregulation of CD14 and upregulation of DC identity and co-stimulatory markers, including CD209, HLA-DR, CD80, CD86, and CD83. To assess the phenotypic status of the cells generated under different O_2_ conditions, we analyzed surface marker expression profiles indicative of this immunophenotypic progression. Our study revealed that the proportion of moDCs expressing CD209 and HLA-DR remained high under both physioxic conditions (Figure 3A), indicating preserved cellular integrity (CD209: control: 99.5%, IQR: 99.1–99.8%; 4% O_2_ D4–6: 99.2%, IQR: 97.9–99.6%; 4% O_2_ D0–6: 93.1%, IQR: 85.7–96.3%; HLA-DR: control: 100.0%, IQR: 99.9–100.0%; 4% O_2_ D4–6: 99.9%, IQR: 99.7–100.0%; 4% O_2_ D0–6: 99.9%, IQR: 99.7–99.9%). However, significant reductions in the expression levels of CD209 (*p* < 0.0001) and HLA-DR (*p* = 0.003), as measured by MFI, were observed under continuous physioxic conditions compared to atmospheric O_2_ levels, with significant differences between physioxic maturation and controls for CD209 (*p* = 0.0104) but not for HLA-DR (Figure 3B).

When examining the maturation markers, we found that the proportion of moDCs expressing CD83 and CD80 was unaffected by physioxia. However, their expression levels were significantly reduced under continuous physioxia compared to atmospheric O_2_ levels (CD83: *p* = 0.0031, CD80: *p* = 0.0009). Additionally, a significant reduction in CD83 expression was observed under physioxic maturation (*p* = 0.0344). CD86 was uniformly expressed across all conditions (control: 99.6%, IQR: 99.3–99.8%; 4% O_2_ D4–6: 98.9%, IQR: 97.8–99.3%; 4% O_2_ D0–6: 99.1%, IQR: 98.5–99.4%; Figure 3A), although expression levels were significantly downregulated under continuous physioxia (*p* = 0.0002), similar to the other maturation markers (Figure 3B).

To further investigate the effect of O_2_ levels on tolerance-associated markers, we assessed the expression of CD40, PD-L1, B7-H3, ILT3, and ILT4 on moDCs cultured under atmospheric O_2_, physioxic maturation (4% O_2_ D4–6), or continuous physioxia (4% O_2_ D0–6). Among the markers tested, only ILT4 showed a statistically significant difference, with expression levels significantly upregulated under continuous physioxia compared to atmospheric O_2_ (*p* = 0.0051; Figure 3C). No significant differences were observed for CD40, PD-L1, B7-H3, or ILT3 (Figure 3C).

### 3.3. Physiological O_2_ Levels During the Generation Period Increased Lactate Secretion but Largely Preserved Mitochondrial Function

**Figure 4:**
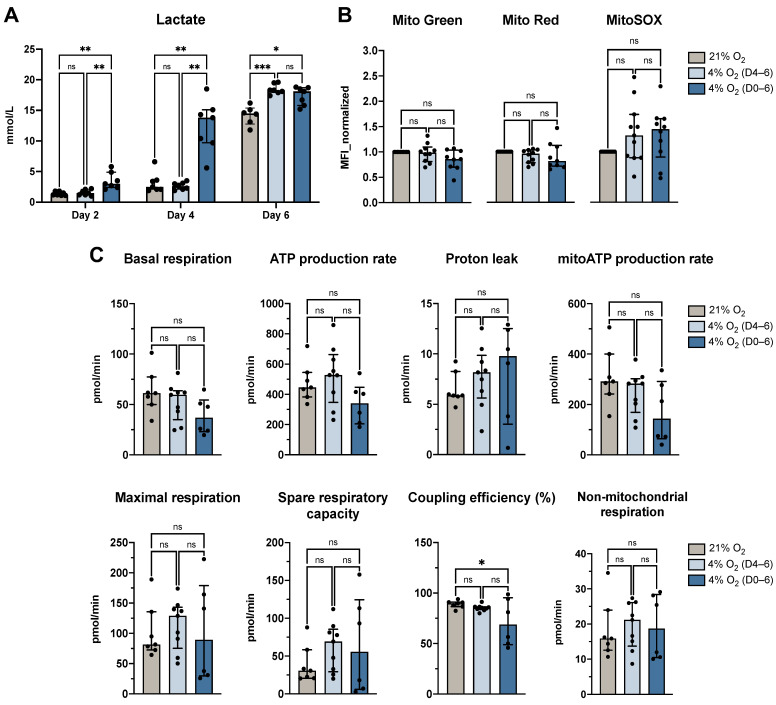
DC generation under 4% O_2_ increased lactate production but did not alter mitochondrial function. (**A**) Lactate production by moDCs (*n* = 7). (**B**) Mitochondrial stainings as indicated by Mitotracker Green (mitochondrial mass), Mitotracker Red (membrane potential), and MitoSOX Red (superoxide production) presented as normalized MFI values (*n* = 11). (**C**) Seahorse Mito Stress Test analysis of moDCs (*n* = 9). Mitochondrial parameters shown include basal respiration (OCR under baseline conditions), ATP production rate (portion of OCR used for ATP synthesis, inferred from the drop after oligomycin addition), proton leak (non-ATP-linked OCR following ATP synthase inhibition), mitoATP production rate (absolute ATP output via oxidative phosphorylation), maximal respiration (peak OCR following FCCP addition), spare respiratory capacity (difference between maximal and basal respiration), coupling efficiency (proportion of basal OCR coupled to ATP production), and non-mitochondrial respiration (residual OCR after inhibition of mitochondrial complexes I and III by rotenone and antimycin A). All cells were cultured under atmospheric O_2_ levels, 4% O_2_ (D4–6), or 4% O_2_ (D0–6). Data are shown as median with interquartile range. Statistical analyses were performed using a mixed-effects model with the Geisser-Greenhouse correction (**A**), an ordinary one-way ANOVA ((**B**): Mito Green, Mito Red; (**C**): basal respiration, ATP production rate, proton leak, mitoATP production rate, maximal respiration, coupling efficiency), or the Kruskal-Wallis test with Dunn’s post-hoc analysis ((**B**): MitoSOX; (**C**): spare respiratory capacity, non-mitochondrial respiration). Statistical significance is denoted by *, where * represents *p* < 0.05, ** represents *p* < 0.01, and *** represents *p* < 0.001. Abbreviations used: O_2_, oxygen; D0–6, days 0 to 6; D4–6, days 4 to 6; moDCs, monocyte-derived dendritic cells; MFI, mean fluorescence intensity; ANOVA, analysis of variance; OCR, oxygen consumption rate; ATP, adenosine triphosphate; FCCP, carbonyl cyanide-p-trifluoromethoxyphenylhydrazone; ns, not significant.

To assess the impact of physiological O_2_ levels on DC metabolism, lactate secretion and mitochondrial parameters were evaluated under atmospheric (21% O_2_) and physioxic (4% O_2_) conditions. While glycolytic activity, as indicated by lactate production, was elevated under physioxia, mitochondrial function remained mostly unaffected (Figure 4).

Lactate measurements revealed a significant increase in secretion by moDCs cultured under continuous physioxia compared to atmospheric O_2_ levels on day 2 (*p* = 0.0063) and day 4 (*p* = 0.0023; Figure 4A). By day 6, lactate levels were significantly higher in both physioxic culture conditions compared to control cells (D4–6: *p* = 0.0002, D0–6: *p* = 0.0163).

Mitochondrial characteristics were assessed using fluorescent dyes (Mitotracker Green, Red, and MitoSOX Red) and Seahorse metabolic flux analysis. Fluorescence-based measurements indicated no significant differences in mitochondrial mass, membrane potential, or mitochondrial superoxide levels between atmospheric and physioxic conditions (Figure 4B), suggesting preserved mitochondrial integrity under both conditions. In parallel, the Seahorse Mito Stress Test evaluated parameters including basal respiration, ATP production rate, proton leak, maximal respiration, spare respiratory capacity, and non-mitochondrial respiration. Most parameters showed no significant differences between physioxic and normoxic culture conditions, aligning with the fluorescence-based findings. Notably, a modest but statistically significant decrease in coupling efficiency was observed under continuous physioxia compared to atmospheric O_2_ (*p* = 0.0489; Figure 4C), indicating reduced efficiency in ATP production relative to total O_2_ consumption. Although there was an observed trend towards lower basal respiration and mitochondrial ATP production rate under physioxic conditions, these differences were not statistically significant. Together, these results suggest that while physioxia may slightly reduce respiratory efficiency, mitochondrial health remains uncompromised, and any shifts in ATP production are minimal.

### 3.4. O_2_ Levels During the Allo-MLR Modulate T Cell Responses

**Figure 5:**
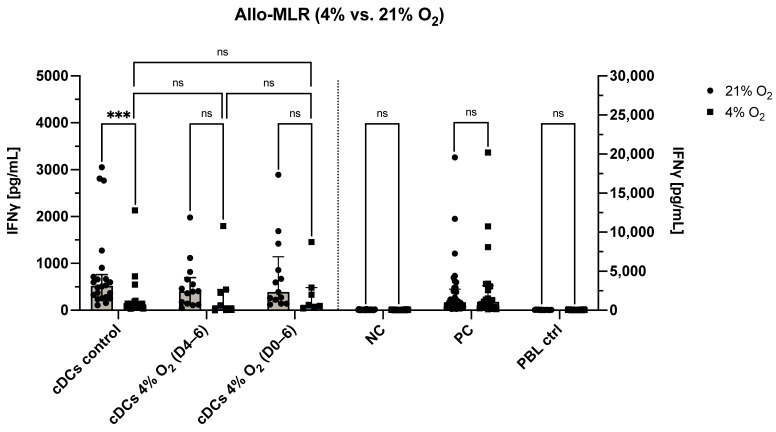
O_2_ levels during the allo-MLR modulate T cell responses. IFN-γ secretion (pg/mL) by PBLs stimulated with control moDCs (cultured at 21% O_2_), moDCs exposed to physioxia during maturation (4% O_2_ D4–6), or moDCs cultured under continuous physioxia (4% O_2_ D0–6), as well as NC, PC (PHA-stimulated PBLs), and PBL-only control (autologous PBLs), in 5-day allo-MLRs conducted at either 21% or 4% O_2_. Data are shown as median with interquartile range. Comparisons for the three moDC conditions were performed using the Wilcoxon matched-pairs signed rank test. Controls were analyzed using a mixed-effects model with Geisser-Greenhouse correction and Šídák’s multiple comparisons test. Statistical significance is denoted by *, where *** represents *p* < 0.001. Abbreviations used: O_2_, oxygen; moDCs, monocyte-derived dendritic cells; D0–6, days 0 to 6; D4–6, days 4 to 6; allo-MLR, allogeneic mixed lymphocyte reaction; IFN-γ, interferon-gamma; PBLs, peripheral blood lymphocytes; NC, negative control; PC, positive control; PHA, phytohemagglutinin; ns, not significant.

**Figure 6:**
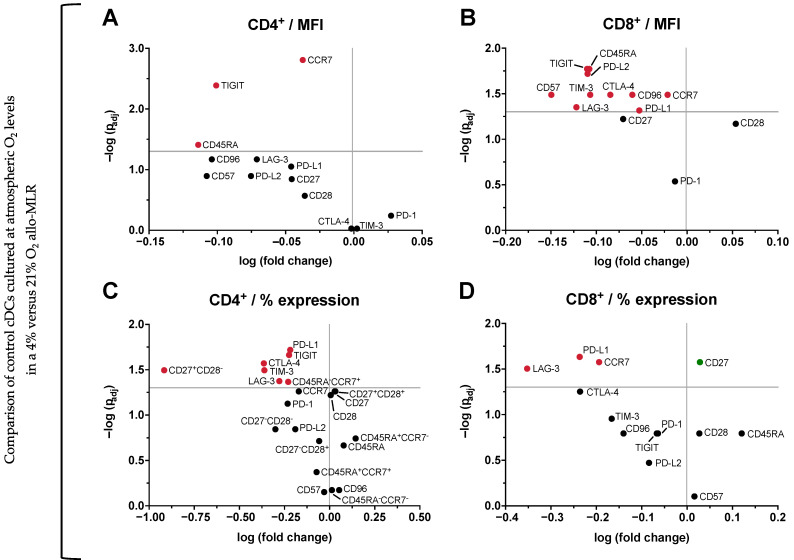
O_2_ levels during the allo-MLR modulate T cell phenotypes. Phenotypic analysis of CD4⁺ and CD8⁺ T cell subsets was performed on day 5 of the allo-MLR to evaluate the impact of O_2_ tension during T cell activation. Control moDCs cultured at atmospheric O_2_ levels were used to stimulate PBLs in either 4% or 21% O_2_ allo-MLRs. Significant changes reflect the effect of physioxia (4% O_2_) compared to atmospheric conditions (21% O_2_). Values represent either MFI (**A**,**B**) or percentage of expression (**C**,**D**) in CD4⁺ or CD8⁺ T cell subsets. Data are presented as −log (Benjamini-Hochberg-adjusted *p*-value; derived from two-tailed paired *t*-tests) versus log (fold change). Statistically significant differences (*p* < 0.05) are highlighted in red (downregulation) or green (upregulation) (*n* = 7). Abbreviations used: O_2_, oxygen; moDCs, monocyte-derived dendritic cells; D0–6, days 0 to 6; D4–6, days 4 to 6; allo-MLR, allogeneic mixed lymphocyte reaction; PBLs, peripheral blood lymphocytes; CD, cluster of differentiation; MFI, mean fluorescence intensity; TIGIT, T cell immunoreceptor with Ig and ITIM domains; CCR7, C-C chemokine receptor type 7; LAG-3, lymphocyte activation gene 3; PD-L1/2, programmed death-ligand 1/2; CTLA-4, cytotoxic T-lymphocyte-associated protein 4; TIM-3, T cell immunoglobulin and mucin-domain containing-3; PD-1, programmed cell death protein 1.

To investigate the influence of physiological O_2_ levels on DC function in terms of their capacity to stimulate allogeneic T cell responses, we conducted mixed lymphocyte reactions (allo-MLR) under both atmospheric (21% O_2_) and physioxic (4% O_2_) conditions, evaluating IFN-γ secretion and phenotypic changes of peripheral blood lymphocytes (PBLs) after five days of co-culture (Figure 5 and Figure 6). 

As a first step, the effect of O_2_ levels during the allo-MLR itself was assessed. DCs cultured under atmospheric O_2_ were co-cultured with allogeneic PBLs in either a 21% or 4% O_2_ allo-MLR. Under physioxic allo-MLR conditions, these control DCs induced significantly lower levels of IFN-γ secretion compared to atmospheric conditions (*p* < 0.0001), suggesting that low O_2_ leads to T cell hyporesponsiveness in this context (Figure 5). In contrast, no significant differences in IFN-γ secretion were observed when comparing 21% and 4% O_2_ allo-MLRs for any of the following: negative control, positive control, PBL-only control, moDCs matured under physioxia (4% O_2_ D4–6), or moDCs cultured under continuous physioxia (4% O_2_ D0–6). These findings suggest that the reduced IFN-γ levels seen under physioxia may be specific to the combination of atmospheric moDCs and low O_2_ during T cell activation, rather than a general suppression of PBL responsiveness under physioxic conditions.

Next, the impact of O_2_ exposure during DC generation was examined under physioxic allo-MLR conditions. Specifically, IFN-γ secretion was compared across all three DC culture conditions—atmospheric (21% O_2_), physioxic maturation (4% O_2_ D4–6), and continuous physioxia (4% O_2_ D0–6)—in a 4% O_2_ allo-MLR. In this setting, no significant differences were observed between the groups (Figure 5), suggesting that preconditioning DCs under physioxia does not markedly alter their IFN-γ-inducing capacity in allogeneic settings.

To gain further insights into how O_2_ tension affects T cell phenotypes beyond IFN-γ secretion, flow cytometric profiling was performed on PBLs after five days of allo-MLR. For this, cells were analyzed for memory and exhaustion markers on CD4⁺ and CD8⁺ subsets.

As a baseline comparison, PBLs co-cultured with control DCs (cultured entirely under atmospheric conditions) were analyzed after allo-MLR under either 21% or 4% O_2_. In the CD4⁺ population, significant reductions in MFI were observed for CCR7, TIGIT, and CD45RA under physioxia (Figure 6A). In the CD8⁺ population, MFI of CD57, TIGIT, CD45RA, PD-L2, TIM-3, LAG-3, CTLA-4, CD96, PD-L1, and CCR7 were all significantly decreased under physioxic conditions (Figure 6B). In terms of percentage marker expression, CD4⁺ cells showed significantly reduced frequencies of CD27⁺CD28⁻, PD-L1⁺, TIGIT⁺, CTLA-4⁺, TIM-3⁺, LAG-3⁺, and CD45RA⁻CCR7⁺ cells (central memory T cells), while CD8⁺ cells showed a decrease in LAG-3⁺, PD-L1⁺, and CCR7⁺ cells, and a corresponding increase in CD27⁺ cells (Figure 6C,D). The control conditions (NC, PC, PBL-only) exhibited minor changes in this setup, limited to MFI shifts in a few markers without significant alterations in the percentage of marker-expressing cells (Figure S3), in line with the IFN-γ secretion data.

Next, the influence of O_2_ exposure during DC maturation was assessed. When comparing DCs exposed to physioxia during maturation (4% O_2_ D4–6) to control DCs (21% O_2_) in a 4% O_2_ allo-MLR, no significant differences were observed in the expression of any markers on either CD4^+^ or CD8^+^ T cells.

Finally, the effects of continuous physioxia (4% O_2_ D0–6) were assessed relative to atmospheric DCs in a 4% O_2_ allo-MLR. For CD4⁺ cells, only CD45RA showed a significant reduction in MFI upon physioxic preconditioning (*p* = 0.034), while no significant differences were observed in CD8^+^ cells. Marker expression frequencies remained unchanged in both T cell subsets.

These results reveal broad downregulation of key exhaustion and memory markers across both CD4⁺ and CD8⁺ T cell subsets along with reduced IFN-γ secretion in response to physioxic allo-MLRs. On the other hand, preconditioning DCs under physioxia alone had limited impact on T cell phenotypes and IFN-γ secretion levels.

## 4. Discussion

Understanding how O_2_ levels influence both DC phenotype and their downstream T cell interactions is crucial for designing more effective immunotherapies and for studying tolerance mechanisms in transplantation and autoimmunity. While most in vitro studies rely on atmospheric O_2_ levels (21%), these are far from the physiological conditions that DCs encounter in vivo. Previous research has predominantly focused on exploring DC biology in pathological hypoxia (<2% O_2_), relevant for cancer, bacterial infections, arthritis, wound healing, and inflammatory lesions [49]. However, little is known about how physiological O_2_ levels affect immune homeostasis. DCs continuously sample antigens in the steady state but avoid full maturation unless activated by PAMPs or DAMPs. The shift from tolerogenic to immunogenic DCs occurs upon encountering inflammatory signals, ensuring that immune responses are mounted only in the presence of a true threat. Understanding the tolerogenic role of DCs under homeostatic conditions has significant implications. Dysregulation of tolerogenic DCs can lead to loss of tolerance and autoimmunity. In addition, tumor microenvironments often exploit tolerogenic mechanisms to suppress DC function and evade immune responses. The main goal of this study was to understand how homeostatic immune responses are generated by mimicking the in vivo conditions of physiological O_2_ levels (4% O_2_) and comparing them to hyperoxia (21% O_2_) typically present in in vitro cell culture. We demonstrated that culturing ex vivo-generated moDCs under physiological O_2_ levels leads to reduced monocyte-to-mDC transformation rate and programs the resulting cells toward a tolerogenic phenotype, characterized by increased secretion of lactate, a semi-mature surface marker profile, and altered T cell priming. This suggests a critical role for O_2_ in maintaining DC homeostasis, with potential implications for both steady-state and pathological conditions. The observed effects were most pronounced when physioxia was applied throughout the entire culture period, while exposure during the maturation phase only had subtler effects. In vivo, DCs can differentiate from monocytes in inflammatory conditions often characterized by hypoxia [29]. Our experimental setting using physioxia is intended to mimic a homeostatic or steady-state environment. The observed reduction in monocyte-to-mDC transformation rate under physioxia suggests that the metabolic demands in this condition may limit monocyte commitment to the DC lineage in the absence of inflammatory cues, aligning with the physiological scenario under non-inflammatory conditions. The observed reduction in viability under physioxia should be carefully interpreted alongside functional outcomes of the viable DC population. Notably, our assessment on day 6 via PI exclusion reflects the total cell pool before distinguishing between differentiated moDCs and remaining monocytes based on extensive phenotypic profiling. Thus, physioxia affected both overall cell survival and monocyte-to-DC transformation, the latter confirmed by DC lineage markers. This raises the question of why viability decreased under conditions that were intended to mimic physiological O_2_ levels. Monocytes were isolated from human blood under standard atmospheric conditions and were then immediately cultured under physioxia. This shift does not mirror the gradual or tissue-specific O_2_ exposure that cells would experience in vivo and highlights the difficulty of mimicking physiological conditions in vitro.

Interestingly, while the percentage of cells expressing key surface molecules remained high (CD209, HLA-DR, CD86) or unchanged (CD80, CD83) under physioxia, their expression levels (as indicated by MFI) were significantly reduced. This discrepancy suggests that although DC identity was preserved, the density of the marker expression on the cell surface declined, which indicates a collective impairment of DC-mediated T cell activation by weakening of co-stimulation (CD86, CD80, CD83), antigen presentation (HLA-DR), and antigen uptake (CD209). Of note, phenotypic profiling was performed on the total viable cell population, capturing not only differentiated DCs but also cells that did not fully commit to the DC lineage. As such, the observed shifts reflect both phenotypic modulation of DCs and impaired monocyte-to-mDC transformation efficiency under physioxia.

The next step for homeostatic DCs in vivo is their potential interaction with T cells under steady-state conditions, where tissue O_2_ levels are considerably lower than atmospheric. To investigate their functionality in this context, we assessed the capacity of physioxia-conditioned DCs to activate allogeneic T cells under reduced O_2_ conditions. We observed no differences in the ability of physioxia-conditioned or atmospheric DCs to induce IFN-γ secretion or to alter the phenotype of PBLs under these conditions. This may suggest that physiological O_2_ levels alone do not significantly contribute to shaping immune homeostasis—though the complexity of in vivo interactions cannot be fully recapitulated in this system. However, when we introduced a shift from 21% to 4% O_2_ during the T cell activation phase, IFN-γ secretion was reduced in control moDC co-cultures. In parallel, we observed a broad downregulation of markers associated with co-inhibitory signaling, memory differentiation, and effector maturation across both CD4^+^ and CD8^+^ subsets. This suggests that low O_2_ levels at the site of T cell priming may impair effector cytokine production and modulate the quality of T cell responses. Importantly, no difference in IFN-γ secretion was observed between 21% and 4% O_2_ in the absence of DCs, highlighting that the O_2_ levels did not have a direct effect on T cell function itself but rather attenuated the immunostimulatory capacity of ex vivo-generated DCs during their interaction with T cells. Rather than indicating a classical exhausted phenotype, which would typically involve upregulation of checkpoint molecules and co-inhibitory receptors, the observed profile suggests a hyporesponsive or metabolically restrained T cell state. This is characterized by insufficient activation to induce either effector function or regulatory feedback mechanisms. Markers involved in terminal differentiation (CD57) and lymphoid homing and memory formation (CCR7, CD45RA) were likewise reduced, along with several immune checkpoints and regulatory receptors (PD-1, PD-L1, PD-L2, TIGIT, TIM-3, CTLA-4, LAG-3, CD96). Importantly, our results highlight that immune responses cannot be defined by the absence or presence of individual markers alone but must be instead understood as an integrated functional and phenotypic state. It is important to note that this experimental setup, in which DCs are generated under atmospheric O_2_ and T cells are activated under physioxic conditions, does not reflect a physiological scenario, as both cell types would naturally co-localize within the same O_2_ microenvironment in vivo. These insights have implications for immunotherapeutic applications, where ex vivo-generated DCs are administered into low-O_2_ tissue environments. Atmospheric O_2_ levels are standard during in vitro quality assessments of such cell products, which may not reflect their functional potential in vivo. Our results indicate that physioxia shapes the DC-T cell crosstalk—both in terms of inflammatory cytokine output and T cell phenotypic profile—in a way that does not support robust effector responses. This highlights the need to consider O_2_ as a critical environmental regulator of immune activation thresholds.

To better understand the mechanisms underlying the induction of a tolerogenic state under physioxia, we examined cellular metabolism as a potential driver of this phenotype. Immunometabolism is increasingly recognized as a key regulator of immune cell fate, not just in terms of energy production, but also through the integration of environmental signals into functional outcomes. As outlined in our recent review, shifts in cellular respiration stemming from the surrounding O_2_ environment can influence whether DCs adopt an immunogenic or tolerogenic profile [49]. This affects features such as cytokine secretion, antigen presentation, and T cell priming. On the metabolic level, we observed that physioxia enhanced lactate production, consistent with a shift toward aerobic glycolysis—also known as the Warburg effect [51]—a hallmark associated with tolerogenic DCs. This metabolite has been shown to influence immune regulation by promoting tolerogenic DC functions, such as the induction of regulatory T cells and suppression of pro-inflammatory cytokines [52,53,54,55,56]. Despite elevated lactate levels, mitochondrial integrity and respiration remained largely intact in physioxia-conditioned DCs. This suggests a metabolic rewiring rather than a collapse of mitochondrial function and may act in concert with the downregulation of co-stimulatory surface molecules, altered cytokine secretion pattern, and changes in antigen uptake or processing capacity to maintain a non-immunogenic profile under steady-state conditions.

Our findings contrast with a study by Futalan et al. (2011), which reported that generation under 5% O_2_ did not influence yield, phenotype, or T cell activation of moDCs [57]. Several factors may explain this discrepancy. First, the O_2_ level in our study was slightly lower (4% O_2_), which may have crossed a functional threshold affecting cellular metabolism and protein expression. Second, the cell isolation methods differed: while we used CD14^+^ immunomagnetic separation to enrich for monocytes, Futalan et al. used adherence-based selection, which may yield a more heterogeneous starting population and influence subsequent differentiation. Furthermore, differences in maturation stimuli, although similar in composition, could have further contributed to the divergence in results. Lastly, our study maintained tighter control of O_2_ levels throughout the entire experimental setup and also compared different O_2_ levels during the allo-MLR, whereas Futalan et al. performed their co-cultures under atmospheric O_2_ conditions. These subtle yet important distinctions highlight the sensitivity of DC generation to microenvironmental parameters and underscore the need for standardization when comparing studies using physiological O_2_ levels.

In summary, DCs are central regulators of immune homeostasis, balancing tolerance and immunity through their interaction with T cells and their response to microenvironmental cues. The tolerogenic phenotype of DCs under homeostatic conditions is influenced by cytokines, metabolic signals, and O_2_ levels. Our findings point toward a potential link between O_2_-sensitive metabolic reprogramming and the establishment of a semi-mature, tolerogenic DC state by limiting robust inflammatory activation, advancing our understanding of immune regulation in health and disease. Physiological O_2_ levels may act as a stabilizing factor in steady-state conditions, whereas the hyperoxic conditions typically used in in vitro studies may drive DCs toward an inflammatory state, potentially misrepresenting their behavior in vivo. Recognizing atmospheric O_2_ as a state that may drive immunogenicity offers a valuable perspective for future studies seeking to bridge the gap between in vitro models and in vivo immune regulation. Future research should ensure that both DCs and their interacting immune cells are studied under physiologically relevant O_2_ conditions to more accurately reflect in vivo scenarios.

## Data Availability

The data presented in this study are available on request from the corresponding author.

## Supplementary Materials

The following supporting information can be downloaded at: https://www.mdpi.com/article/10.3390/cells14100736/s1, Table S1: Antibody concentrations; Figure S1: Representative gating strategy for analysis of DC phenotype; Figure S2: Representative gating strategy for analysis of PBL phenotype; Figure S3: Phenotypic analysis of CD4⁺ and CD8⁺ T cell subsets on day 5 of the allo-MLR under the three control conditions.

## Abbreviations

allo-MLR	Allogeneic mixed lymphocyte reaction
ATP	Adenosine triphosphate
BSA	Bovine serum albumin
CCR7	C-C chemokine receptor 7
CD	Cluster of differentiation
moDCs	Monocyte-derived dendritic cells
CO_2_	Carbon dioxide
CTLA-4	Cytotoxic T-lymphocyte-associated protein 4
DAMPs	Damage-associated molecular patterns
DCs	Dendritic cells
DMSO	Dimethyl sulfoxide
FBS	Fetal bovine serum
FCCP	Carbonyl cyanide-p-trifluoromethoxyphenylhydrazone
FMO	Fluorescence-minus-one
FSC	Forward scatter
GM-CSF	Granulocyte-macrophage colony-stimulating factor
hAB	Human AB serum
HLA-DR	Human leukocyte antigen-DR
iDCs	Immature dendritic cells
IL	Interleukin
ILT	Immunoglobulin-like transcript
IFN-γ	Interferon-gamma
IQR	Interquartile range
LAG-3	Lymphocyte activation gene 3
mDCs	Mature dendritic cells
MFI	Mean fluorescence intensity
moDCs	Monocyte-derived dendritic cells
ns	Not significant
O_2_	Oxygen
OCR	Oxygen consumption rate
PAMPs	Pathogen-associated molecular patterns
PBLs	Peripheral blood lymphocytes
PBS	Phosphate-buffered saline
PD-1	Programmed death-1
PD-L1/L2	Programmed death-ligand 1/2
PDL	Poly-D-lysine
PGE_2_	Prostaglandin E_2_
PI	Propidium iodide
SSC	Side scatter
Th	T helper cell subset
TIGIT	T cell immunoreceptor with immunoglobulin and ITIM domain
TIGIT	T cell immunoreceptor with immunoglobulin and ITIM domain
TNF	Tumor necrosis factor
Tregs	Regulatory T cells
