# Supplementary material for: Physiological Oxygen Levels in the Microenvironment Program Ex Vivo-Generated Conventional Dendritic Cells Toward a Tolerogenic Phenotype
Source: Cells. 2025 May 18;14(10):736. doi: 10.3390/cells14100736 (PMC12110723; doi:10.3390/cells14100736)
Supplement: Supplementary file 1 [file cells-14-00736-s001.zip › cells-3598749-supplementary.pdf]

Table S1. Antibody concentrations. Overview of all antibodies and functional dyes used for flow cytometric analysis. The table includes the target antigen, fluorochrome, manufacturer, product number, and the final amount used per test. Fluorescent viability dyes and mitochondrial probes are also listed with their working concentrations. Abbreviations used: APC, allophycocyanin; BV, Brilliant Violet; Cy, cyanine; FITC, fluorescein isothiocyanate; PE, phycoerythrin; PerCP, peridinin-chlorophyll-protein; FM, formyl methyl; CMXRos, chloromethyl-X-rosamine; CD, cluster of differentiation; HLA-DR, human leukocyte antigen – DR isotype; PD-L1, programmed death-ligand 1; PD-L2, programmed death-ligand 2; PD-1, programmed cell death protein 1; CTLA-4, cytotoxic T-lymphocyte-associated protein 4; TIM-3, T cell immunoglobulin and mucin-domain containing-3; LAG-3, lymphocyte activation gene 3; TIGIT, T cell immunoreceptor with Ig and ITIM domains; B7-H3, B7 homolog 3; ILT, immunoglobulin-like transcript.

**Antibody**	**Manufacturer & product number**	**Amount used per test**
PE/Cyanine7 anti-human CD209 (DC-SIGN)	BioLegend, 330114	100 ng
PE/Cyanine7 Mouse IgG2a, κ Isotype Ctrl	BioLegend, 400232	100 ng
KIRAVIA Blue 520 anti-human HLA-DR	BioLegend, 307679	200 ng
KIRAVIA Blue 520 Mouse IgG2a, κ Isotype Ctrl	BioLegend, 400278	200 ng
Brilliant Violet 711 anti-human CD14	BioLegend, 301838	75 ng
Brilliant Violet 711 Mouse IgG2a, κ Isotype Ctrl	BioLegend, 400272	75 ng
Brilliant Violet 785 anti-human CD83	BioLegend, 305338	600 ng
Brilliant Violet 785 Mouse IgG1, κ Isotype Ctrl	BioLegend, 400170	600 ng
Brilliant Violet 605 anti-human CD86	BioLegend, 374214	300 ng
Brilliant Violet 605 Mouse IgG1, κ Isotype Ctrl	BioLegend, 400162	300 ng
APC anti-human CD80	BioLegend, 305220	600 ng
APC Mouse IgG1, κ Isotype Ctrl	BioLegend, 400120	600 ng
PE/Dazzle 594 anti-human CD276 (B7-H3)	BioLegend, 351012	400 ng
Brilliant Violet 711 anti-human CD274 (B7-H1, PD-L1)	BioLegend, 329722	200 ng
BV650 anti-human CD40	BioLegend, 334338	150 ng
APC anti-human CD85d (ILT4)	BioLegend, 338708	100 ng
PE anti-human CD85k (ILT3)	BioLegend, 333007	200 ng
PE anti-human CD209 (DC SIGN)	BioLegend, 330106	100 ng
PerCP/Cy5.5 anti-human CD11c	BioLegend, 301624	1 µg
Live/Dead Fixable Near-IR Dead Cell Stain Kit	Thermo Fisher Scientific, L34976	1 µl
Human TruStain FcX	BioLegend, 422302	5 µl
TruStain monocyte blocker	BioLegend, 426102	5 µl
FITC anti-human CD4	BioLegend, 300505	200 ng
Brilliant Violet 605 anti-human CD27	BioLegend, 302829	100 ng
APC anti-human CD28	BioLegend, 302911	50 ng
Pacific Blue anti-human CD45RA	BioLegend, 304123	250 ng
Brilliant Violet 421 anti-human CD273 (B7-DC, PD-L2)	BioLegend, 329615	100 ng
APC anti-human CD274 (B7-H1, PD-L1)	BioLegend, 329707	200 ng
PE/Cyanine7 anti-human CD279 (PD-1)	BioLegend, 329917	100 ng
PE anti-human CD96 (TACTILE)	BioLegend, 338406	400 ng
Brilliant Violet 711 anti-human CD366 (Tim-3)	BioLegend, 345024	300 ng
Brilliant Violet 785 anti-human CD57	BioLegend, 393330	300 ng
PE/Cyanine7 anti-human CD223 (LAG-3)	BioLegend, 369310	300 ng
Brilliant Violet 785 anti-human CD152 (CTLA-4)	BioLegend, 369623	300 ng
PE anti-human CCR7	Bio-Techne, FAB197P-100	2 µl (concentration not provided)
Spark Red 718 anti-human CD3	BioLegend, 317362	100 ng
Brilliant Violet 510 anti-human CD8	BioLegend, 344731	50 ng
Brilliant Violet 605 anti-human TIGIT (VSTM3)	BioLegend, 372712	400 ng
Mitotracker Green FM FITC	Thermo Fisher Scientific, M7514	100 nM
Mitotracker Red FM CMXRos PE	Thermo Fisher Scientific, M7512	200 nM
MitoSOX Red PE	Thermo Fisher Scientific, M36008	5 µM

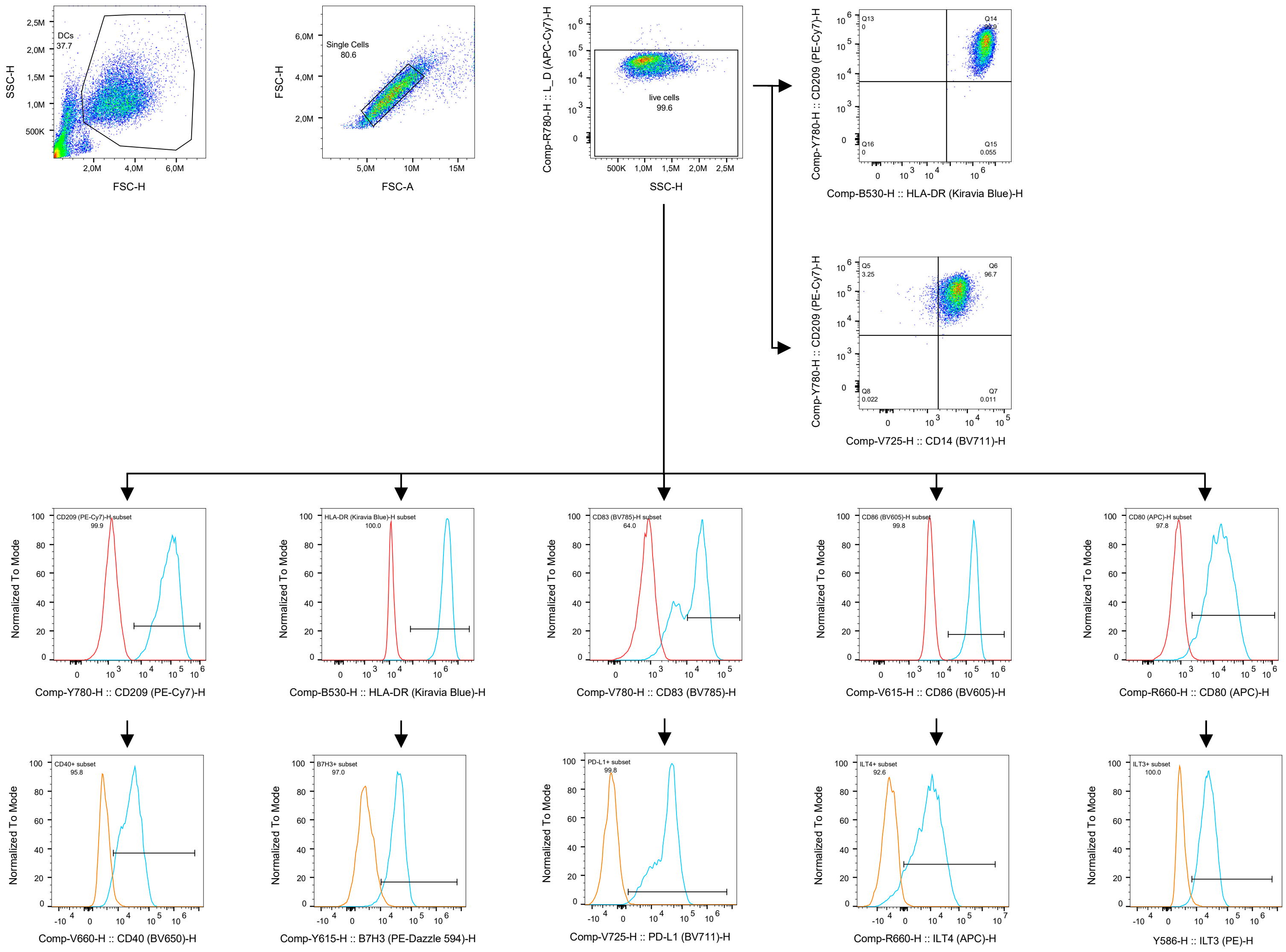

Figure S1. Representative gating strategy for analysis of dendritic cell (DC) phenotype. Cells were first gated based on forward and side scatter (FSC-H vs. SSC-H) to exclude debris, followed by singlet gating (FSC-A vs. FSC-H) and viability staining using the LIVE/DEAD Fixable Near-IR Dead Cell Stain Kit. DC identity was checked by CD209⁺HLA-DR⁺ expression, while the percentage of remaining monocytes (CD209⁻CD14⁺) was assessed using a CD209/CD14 gate. Two separate antibody panels were employed for phenotypic analysis: a maturation-associated DC phenotyping panel (CD209, HLA-DR, CD14, CD83, CD80, CD86), and a tolerance-associated panel (CD40, PD-L1, B7-H3, ILT3, ILT4). Gating for the maturation panel was guided by condition-specific isotype controls (shown in red), except for CD83, where gating was based on a clearly distinguishable positive population. Gating for the tolerance panel was based on fluorescence minus one (FMO) controls (shown in orange). The basic gating strategy (FSC/SSC, singlets, viability) was applied consistently across both panels. Abbreviations used: FSC, forward scatter; SSC, side scatter; DC, dendritic cell; CD, cluster of differentiation; HLA-DR, human leukocyte antigen – DR isotype; PD-L1, programmed death-ligand 1; B7-H3, B7 homolog 3; ILT, immunoglobulin-like transcript.

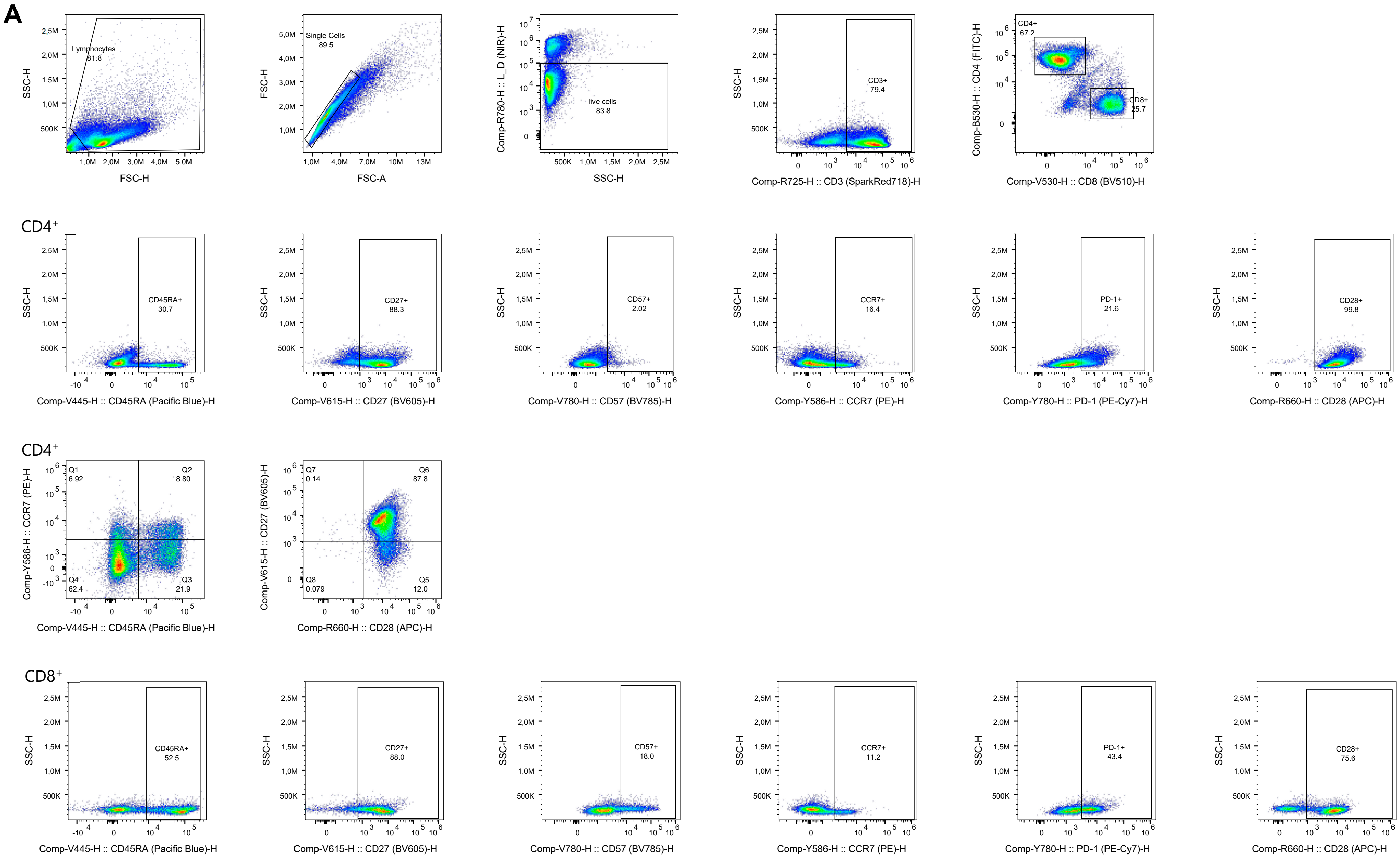

Figure S2 continued on the next page

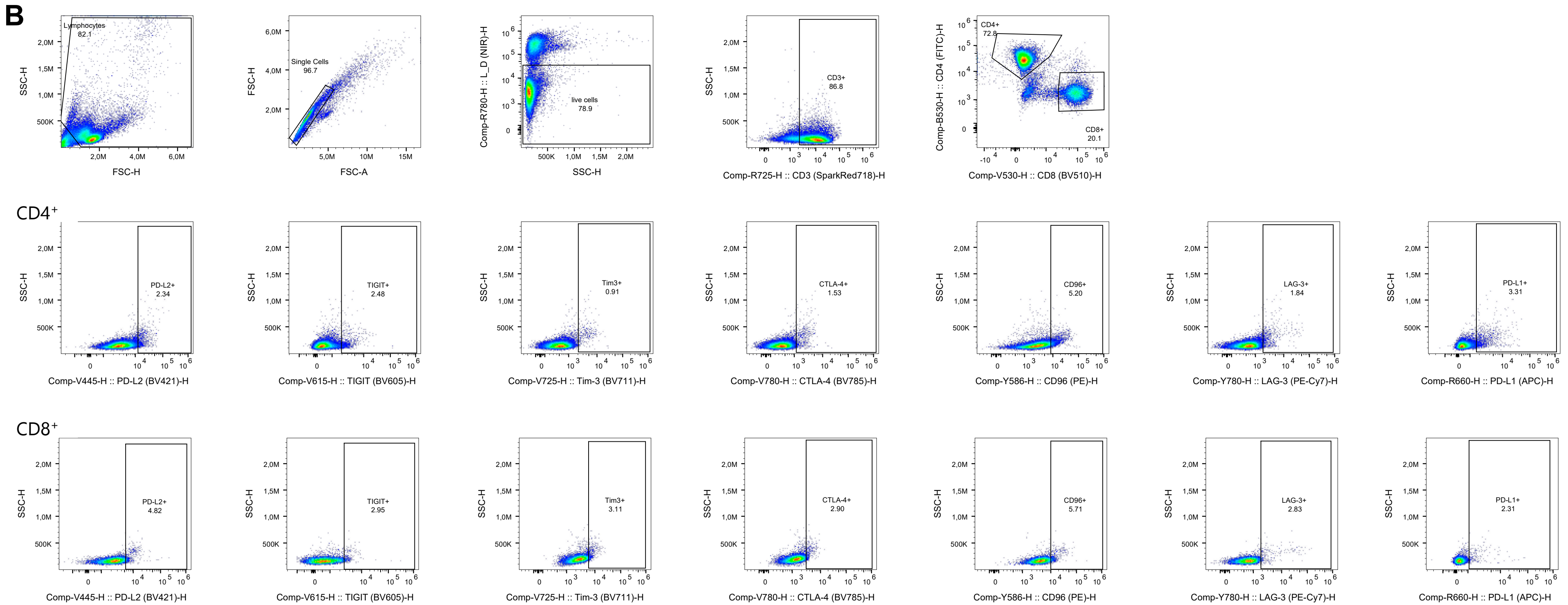

Figure S2. Representative gating strategy for analysis of peripheral blood lymphocyte (PBL) phenotype. Cells were first gated based on forward and side scatter (FSC-H vs. SSC-H) to exclude debris, followed by singlet gating (FSC-A vs. FSC-H) and viability staining using the LIVE/DEAD Fixable Near-IR Dead Cell Stain Kit. $CD3^+$ T cells were identified and further subdivided into $CD4^+$ and $CD8^+$ subsets. Two distinct antibody panels were employed for downstream phenotypic analysis: (**A**) a T cell subsets panel (CD3, CD4, CD8, CD45RA, CCR7, CD27, CD28, CD57, PD-1) and (**B**) a T cell exhaustion panel (CD3, CD4, CD8, PD-L1, PD-L2, TIGIT, TIM-3, CTLA-4, LAG-3, CD96). Gating was based on the negative control condition and adapted where necessary to account for population shifts observed in the experimental samples. For $CD4^+$ T cells, additional subset analyses based on CCR7 and CD45RA expression were used to distinguish between naïve ($CCR7^+CD45RA^+$), central memory ($CCR7^+CD45RA^-$), effector memory ($CCR7^-CD45RA^-$), and effector ($CCR7^-CD45RA^+$) T cells. Classification based on CD28 and CD27 expression was also performed to define subpopulations of memory cells. Abbreviations used: FSC, forward scatter; SSC, side scatter; PBL, peripheral blood lymphocyte; CD, cluster of differentiation; PD-1/PD-L1/PD-L2, programmed death-1/ligands; TIGIT, T cell immunoreceptor with Ig and ITIM domains; TIM-3, T cell immunoglobulin and mucin-domain containing-3; CTLA-4, cytotoxic T-lymphocyte-associated protein 4; LAG-3, lymphocyte activation gene 3; CCR7, C-C chemokine receptor type 7.

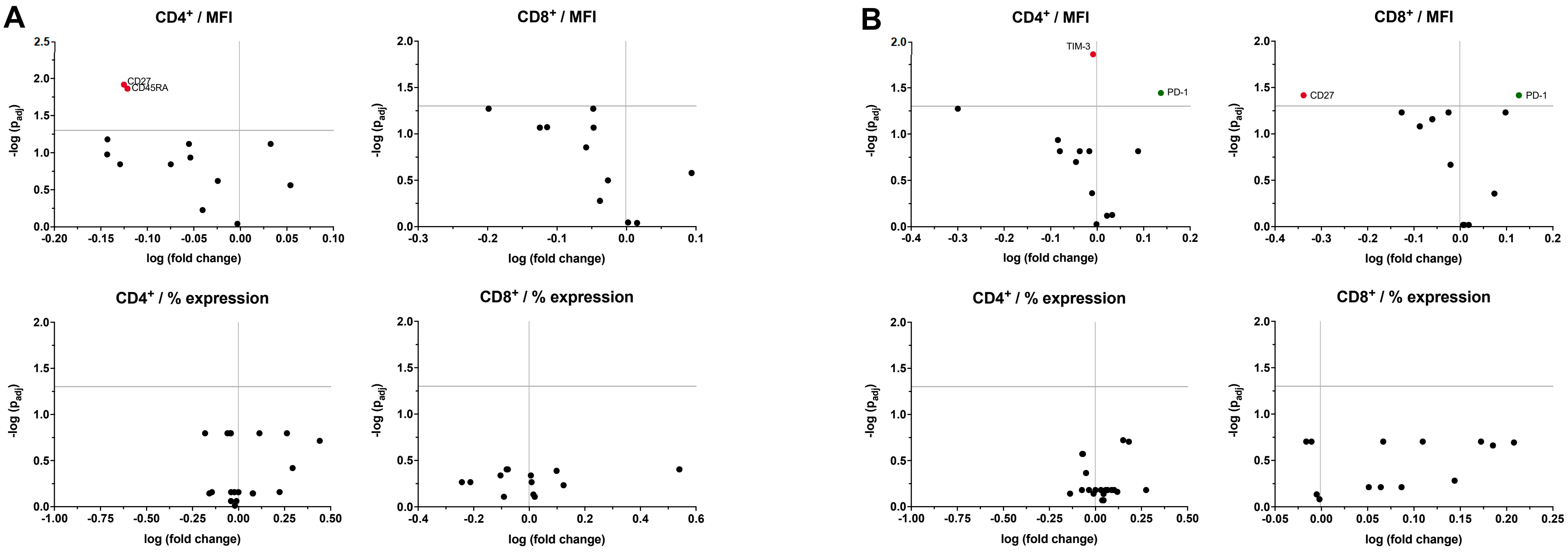

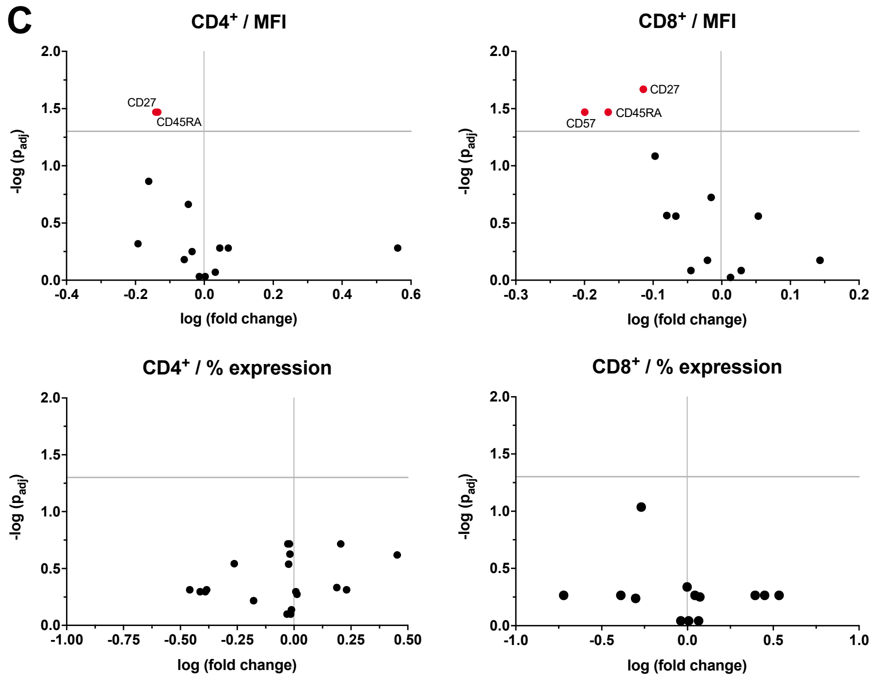

Figure S3. Phenotypic analysis of $CD4^+$ and $CD8^+$ T cell subsets on day 5 of the allo-MLR under the three control conditions: negative control (**A**), positive control (**B**), and PBL-only control (**C**). The same set of markers shown in Fig. 6A-D was analzyed here across all donors; for clarity, only statistically significant changes are labeled in the plots. All comparisons reflect the effect of physioxia (4% O_2) relative to atmospheric conditions (21% O_2). Values represent either MFI or percentage of expression in $CD4^+$ or $CD8^+$ T cell subsets and are displayed as log(fold change) versus -log(Benjamini-Hochberg-adjusted p-value; derived from two-tailed paired *t*-tests). Statistically significant upregulation ($p < 0.05$) is highlighted in green, and downregulation in red ($n = 7$). Abbreviations used: MFI, median fluorescence intensity; CD, cluster of differentiation; TIM-3, T cell immunoglobulin and mucin-domain containing-3; PD-1, programmed cell death protein 1.
